# Developing an Unstructured Supplementary Service Data-based mobile phone app to provide adolescents with sexual reproductive health information: a human-centered design approach

**DOI:** 10.1186/s12874-022-01689-4

**Published:** 2022-08-04

**Authors:** Paul Macharia, Antoni Pérez-Navarro, Irene Inwani, Ruth Nduati, Carme Carrion

**Affiliations:** 1 Faculty of Computer Sciences, Multimedia and Telecommunication, Universitat Oberta de Catalunya, Barcelona, Spain; 2 Consulting in Health Informatics, P.O Box 3966, Nairobi, 00100 Kenya; 3 eHealth Lab Research Group, School of Health Sciences, Universitat Oberta de Catalunya, Barcelona, Spain; 4 Kenyatta National Hospital, Hospital Rd, Nairobi, Kenya; 5 University of Nairobi, University Way, Nairobi, Kenya; 6 eHealth Center, Universitat Oberta de Catalunya, Barcelona, Spain

**Keywords:** Adolescent health, Reproductive health, Mobile phones, Human-centered design

## Abstract

**Background:**

Adolescent pregnancies and sexually-transmitted infections continue to impact 15 – 19-year-olds across the globe. The lack of sexual reproductive health information (SRH) in resource-limited settings due to cultural and societal attitudes towards adolescent SRH could be contributing to the negative outcomes. Innovative approaches, including mobile phone technologies, are needed to address the need for reliable adolescent SRH information.

**Objective:**

The study aimed to co-design a Unstructured Supplementary Service Data (USSD) based mobile app prototype to provide confidential adolescent SRH information on-demand and evaluate the mobile app’s usability and user experience.

**Methods:**

A human-centered design methodology was applied. This practice framework allowed the perspectives and feedback of adolescent users to be included in the iterative design process. To participate, an adolescent must have been 15 to 19 years old, resided in Kibra and would be able to access a mobile phone. Adolescents were enrolled for the alpha and field testing of the app prototype at different time-points. The Mobile Application Rating Scale (MARS) a multidimensional mobile phone evaluation tool was used to access the functionality, engagement, aesthetics and quality of information in the app. Responses from the MARS were reported as mean scores for each category and a mean of the aggregate scores making the app’s quality score. The MARS data was also evaluated as categorical data, A Chi square test of independence was carried out to show significance of any observed differences using cumulative and inverse cumulative distribution functions.

**Results:**

During the usability test, 62/109 (54.9%) of the adolescents that were followed-up had used the app at least once, 30/62 (48.4%) of these were male participants and 32/62 (51.6%) female. On engagement, the app had a mean score of 4.3/5 (SD 0.44), 4.6/5 (SD 0.38) on functionality, 4.3/5 (SD 0.57) on aesthetics and 4.4/5 (SD 0.60) on the quality of information. The overall app quality mean score was 4.4/5 (SD 0.31). The app was described as ‘very interesting’ to use by 44/62 (70.9%) of the participants, 20/44 males and 24/44 females. The content was deemed to be either ‘perfectly’ or ‘well targeted’ on sexual reproductive health by 60/62 (96.7%) adolescents, and the app was rated ‘best app’ by 45/62 (72.6%) adolescents, 27/45 females and 18/45 males, with a *p*-value = 0.011.

**Conclusions:**

Adolescents need on-demand, accurate and trusted SRH information. A mobile phone app is a feasible and acceptable way to deliver adolescent SRH information in resource-limited settings. The USSD mobile phone technology shows promise in the delivery of much needed adolescent SRH information on-demand..

**Supplementary Information:**

The online version contains supplementary material available at 10.1186/s12874-022-01689-4.

## Introduction

Adolescent pregnancies continue to negatively impact girls across the globe, with around 12 million girls aged 15 – 19 years giving birth annually in the developing world [1]. Information on sexual reproductive health [2] and contraception is lacking in majority of resource-limited settings [3]. Although girls bear the most significant impact, boys also lack information and services on reproductive health. Inability to access reproductive health information and services may be a contributing factor to unwanted pregnancies and sexually-transmitted infections among adolescents [4].

The World Health Organization (WHO) considers adolescent pregnancy a public health concern due to its impact on the health of both newborn and mother [5, 6]. In a number of countries, complications arising from giving birth as an adolescent are a leading cause of death [7]. Sexually-transmitted infections among adolescents are increasing, despite the mounting burden on health systems, there has been little research into effective prevention and treatment strategies [8]. Due to the high levels of sexually-transmitted infections among adolescents, there is a need to develop and customize information and educational resources to provide relevant, accessible and up-to-date sexual reproductive health information [9].

Many adolescents access sexual reproductive health information from their peers, parents and technology-based sources, including the social media and internet [10]. Choice of information sources on reproductive health has been found to have a substantial impact on adolescent health outcomes. The large number of unwanted pregnancies, sexually-transmitted infections and mental health related issues indicate current sources don’t meet the adolescents’ information needs [11]. Research has shown that adolescents prefer evidence-based information on sexual reproductive health delivered through innovative approaches. The information should be targeted and adapted to the adolescents’ norms and context [12].

To show how important adolescent reproductive health is as a public health issue, the world health organization (WHO) in 2018 launched a document entitled "WHO recommendations on adolescent sexual and reproductive health and rights”. The document aimed to “provide an overview of sexual and reproductive health and rights issues that may be important for the human rights, health and well-being of adolescents (aged 10–19 years) and the relevant WHO guidelines on how to address them in an easily accessible, user-friendly format".

The human-centered design (HCD) methodology is “an approach to interactive systems development that aims to make systems usable and useful by focusing on the users” [13]. In recent years, the HCD approach to the design of social innovations in global health is on the increase. This is due to the approach focusing on empathy and context in the ideation and iterative design of a health intervention [14]. The HCD lifecycle emphasizes the need to develop a product that meets the needs of the envisioned users [15]. As research shows [16], applying the HCD methodology in the design and development of an mhealth intervention is very important. In our study, three phases of the HCD methodology were applied namely; gathering user needs, content review by subject experts and carrying out alpha and field testing of the app.

In our formative qualitative study to gather the adolescents SRH information needs [17], we identified their current sources, limitations of these sources and the potential role of mobile phone technologies could play in meeting their SRH information needs. Adolescents need information on adolescent-friendly services, sexually-transmitted infections, contraceptives, sexual relationships, abstinence, and drug use. Among mobile phone technologies, the adolescents prefer a technology that enhances privacy, is toll-free, provides information on demand, and works on both feature phones and smartphones [18–20]. The Unstructured Supplementary Service Data (USSD) technology met the adolescents’ user requirement. The technology works in both feature phones and smartphones, nothing is saved on the phone enhancing confidentiality. No installation was required and the services could be provided toll-free.

The USSD is a mobile phone technology that has a similar format to short message services (SMSs). However, USSD offers a messaging service that doesn’t save any data on the user’s device [21]. Already, the USSD technology has been used to enable healthcare workers interact with patients on-demand. For example, in Zimbabwe a resource-limited setting, the USSD technology was used to provide health tips to the general public [22]. In Uganda, the USSD technology was used for health data reporting enhancing accuracy, timeliness and completeness of healthcare data [23].

In this study we aimed to co-design and develop a mobile app prototype to provide adolescents with confidential reproductive health information on demand and evaluate its usability and user experience. The study was guided by a human-centered design approach. 

## Materials and methods

### Mobile phone technology development

A mobile phone app prototype was developed. A human-centered design (HCD) approach guided the iterative process of the mobile app development. The HCD approach is a practice framework that allows users’ perspectives and feedback to be included in the design, development and prototyping of a health intervention [24].

Using the previously identified adolescent sexual reproductive health information needs, content on abstinence, contraceptives, sexually-transmitted infections, sexual relationships and drug use was developed into a paper-based format. This content was then reviewed by 2 adolescent reproductive health experts for its accuracy, relevance, and age appropriateness for the targeted adolescent participants. The 2 experts were I.I. and R.N., researchers with many years of experience working in adolescent reproductive health in Kenya and co-investigators in this study.

The experts reviewed the structure and wording used to ensure information could easily be understood by adolescents. They also ensured that the content by age group was permissible by government policy. For example, it is illegal to provide information on contraceptives to anyone under 18 in Kenya, therefore information on abstinence was made available to participants under 18.

Once the content had been reviewed and agreed upon it was customized into the Echomobile® platform, a telco service provider with presence in Kenya. This provider offers a cloud-based web platform capable of providing a USSD channel to automate personalized communications at scale. The USSD technology is a mobile phone standard that runs on feature phones and smartphones running Android or iOS operating system without any installation, technical development or customization. Only the SRH content was reviewed by content experts and then customized to the USSD platform. On the USSD platform, nothing is saved on the device, all interaction is saved on a backend Echomobile® server. On the other hand, USSD apps can work in Android and iOS and feature phones without installation. However, it is important to note, that Android and iOS are very rare in the target population.

### Study design

A prospective research study design was used to evaluate the usability and user experience of the mobile app prototype. The study was a two-step process: alpha testing and field usability testing. The recruitment, inclusion, exclusion criteria and mobile app access for these two processes is presented in the following sections.

### Participant recruitment

**Fig. 1:**
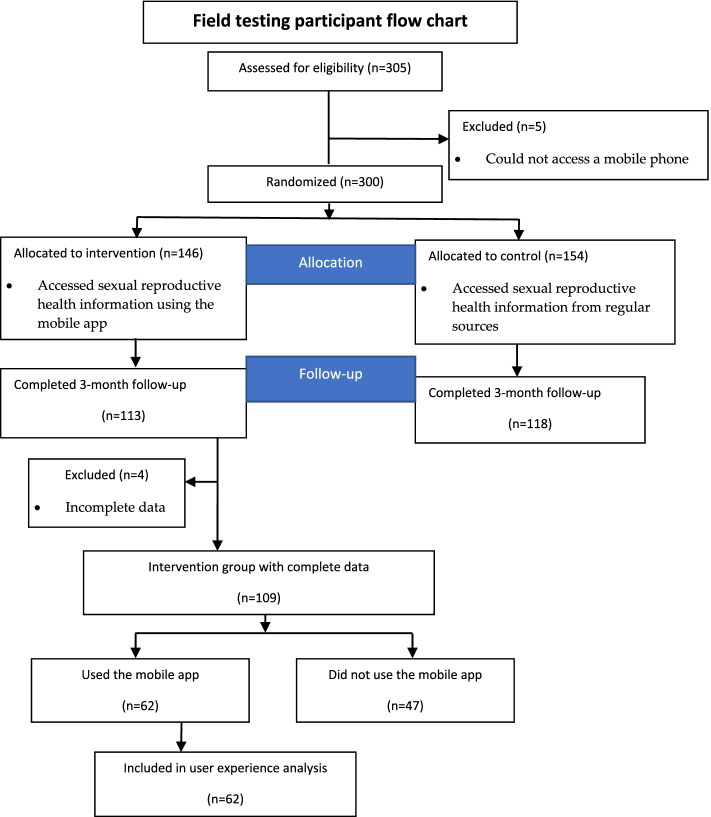
Participant flow chart during field testing

Adolescents were mobilized from the 12 villages in Kibra by two community workers with experience working in youth programs in the area. Kibra is a suburb in the city of Nairobi Kenya with an estimated population of 2.5 million residents. Inter-village ethnic differences exist related to historical, migration and settlement trends. The mobilization targeted events, venues, sport events and other youth programs in the community that attracted 15 – 19-year-olds. Study procedures were explained to all potential participants using a recruitment script, individually or in small groups. Adolescents showing an interest in the study were referred to the recruitment site for screening and potential enrollment. During the alpha testing, all the 38 adolescents mobilized to the study met the eligibility criteria and were enrolled. For the field testing, a total of 305 adolescents were screened and 300 met the eligibility and were enrolled. The Kenya adolescent reproductive health and development policy implementation assessment report projected adolescents aged 15–19 years accessing SRH services to be about 8%. The 8% was used for sample calculation. The sample of 300 was 74 participants more than the minimum sample calculation so that sample strength was still achieved if there was loss to follow-up. For the 300 enrolled, 146 were randomized to use the mobile app for a 3-month period. The adolescents in the intervention group accessed SRH information on the mobile app prototype, nothing was provided to adolescents in the control group. Figure 1 below shows participant enrollment for the field testing.

### Inclusion and exclusion criteria

To be eligible an adolescent must: 1) have lived in Kibra for at least 3 months; 2) be aged between 15 – 19 years; 3) be willing to take part in the study; and 4) have access to a feature phone or smartphone. Based on the adolescent’s age, a written assent or consent was obtained from each participant before study procedures were administered. Both boys and girls meeting the inclusion criteria participated in the study.

### Mobile app access

During both the alpha and field testing, study staff created an account and a user PIN in the USSD app for each participant. Interaction with the USSD app was demonstrated to each adolescent for 5 to 10 min to increase familiarity and understanding of how the app works. Participants could contact the study team through the community mobilizers if they misplaced their PIN or needed any other help using the app. Each participant was offered the phone number of the community mobilizers to call in case they needed guidance on using the USSD app. The USSD app offered a text-based interactivity on the users’ phone. The app also provided contact details of adolescent-friendly healthcare facilities in the Kibra locality so that the adolescent participants could interact with subject experts if they so wished. A video of the USSD app can be availed on request. During the study’s formative stage of gathering user needs, the adolescents indicated they preferred the USSD app content presented in English. The English language is one of the two official languages in Kenya and is taught and used in school for communication.

To access the USSD app, the adolescent participants dialed a 7-digit USSD code on a mobile phone. After dialing the code, the adolescent user was prompted to key-in their user PIN. If the PIN was authenticated, then the user could now access the SRH information. Adolescents could access the USSD app from any location as long as they had their user PIN and their registered mobile phone number on the mobile phone device in use.

### Alpha testing

Initially, 38 participants were recruited for alpha usability testing in the first week of April 2019. Participants were then followed-up at the end of April 2019. A recruitment script was used to explain the study procedures and potential benefits to the adolescents. The alpha usability test, a low-fidelity prototyping of the app [25] was guided by a customized mobile application rating scale (MARS) (Additional file 1: Appendix B). In one published work, the MARS demonstrated excellent internal consistency (α = 0.90) and interrater reliability intra-class correlation coefficient (α = 0.79) [26]. The study team customization only included rephrasing questions to relate to the USSD app and adolescent reproductive health.

The alpha testing evaluated the apps functionality and information content, as well as the usability and user experience of the app. The enrolled participants accessed the app for one month to generate enough data for the alpha usability testing. The MARS was administered at the end of the one month of use.

### Field usability testing

After the app had passed the alpha testing a field usability test was carried out. 109 participants in the intervention group were successfully followed-up, only 62 adolescents had used the app at least once over a 3-month period. Adolescent participants included in the final analysis were the 62 who had used to app. The adolescent participants were enrolled in October 2019 and followed up in December 2019 and January 2020. The field usability testing evaluated the success of the app in providing correct, relevant, and on-demand information, and its usability and user experience. At enrollment, a recruitment script was used to explain the study procedures and potential benefits to the adolescents.

Potential participants were enthusiastic that the mobile phone app would provide accurate, up-to-date information on sexual reproductive health. Each eligible adolescent provided a cellphone number for either their own phone or that of a parent, guardian, or sibling. A customized MARS (Additional file 1: Appendix B) was used at the end of the 3-month period to evaluate the app. Adolescent participants in the alpha testing were eligible for the field testing. To minimize bias, participants were randomly assigned to either using the app or the control group.

### Statistical analysis

Data was analyzed using R software version 3.6.2 [27]. Descriptive statistics were applied to the participant characteristics, mobile application rating scale and user experience evaluation. For application rating scale scores, standard deviation was used to measure the spread. A Chi square test of independence was carried out on the user experience evaluation outcomes. The Chi square test, a non-parametric test is able to provide information on how each group of participants performs. The test shows significance of any observed differences and the categories account for any differences found [28]. The Chi square test of independence was deemed to be the most appropriate for our study. The *p*-value calculation in the study used cumulative distribution functions and inverse cumulative distribution functions [29]. A *p*-value of < 0.05 was deemed to be statistically significant. A Cronbach alpha test [30] was used to provide a measure of the internal consistency of the MARS during the alpha and field testing.

## Results

### The USSD Mobile Phone App

The final paper-based version of the adolescent reproductive health content (Additional file 1: Appendix A) was programmed into the web-based interface provided by Echomobile® for the USSD platform.

**Fig. 2:**
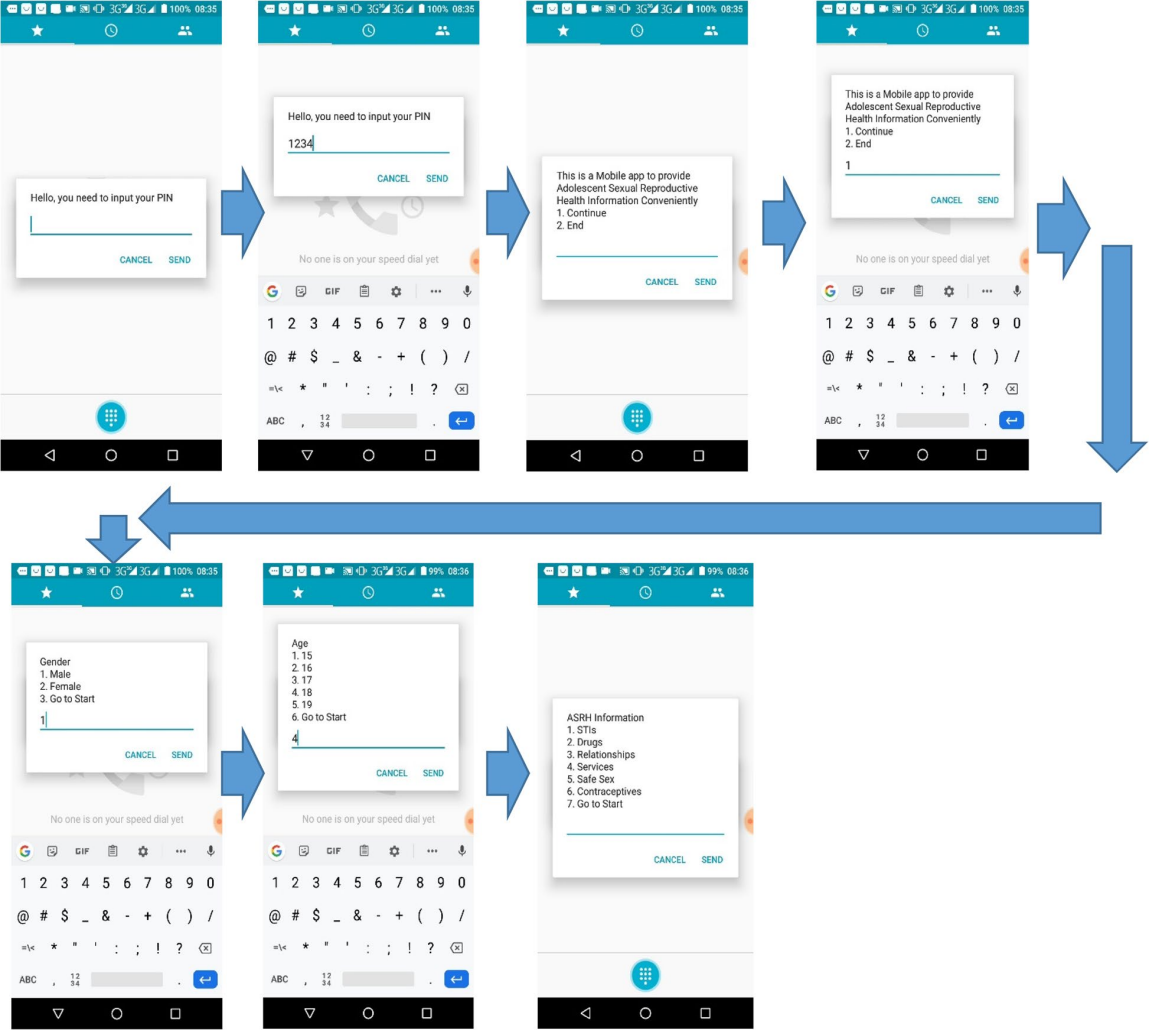
Connecting to the USSD app

To open the app, users dialed a 7-digit code on a mobile phone, which then prompted them to input a pre-assigned PIN linked to their cellphone number. When the users were authenticated, they selected their gender and age on subsequent screens before selecting a sexual reproductive health topic of interest. The users then interacted with screen-by-screen content guided by their input. A selection of the mobile app interactive screens is shown on Fig. 2.

**Fig. 3:**
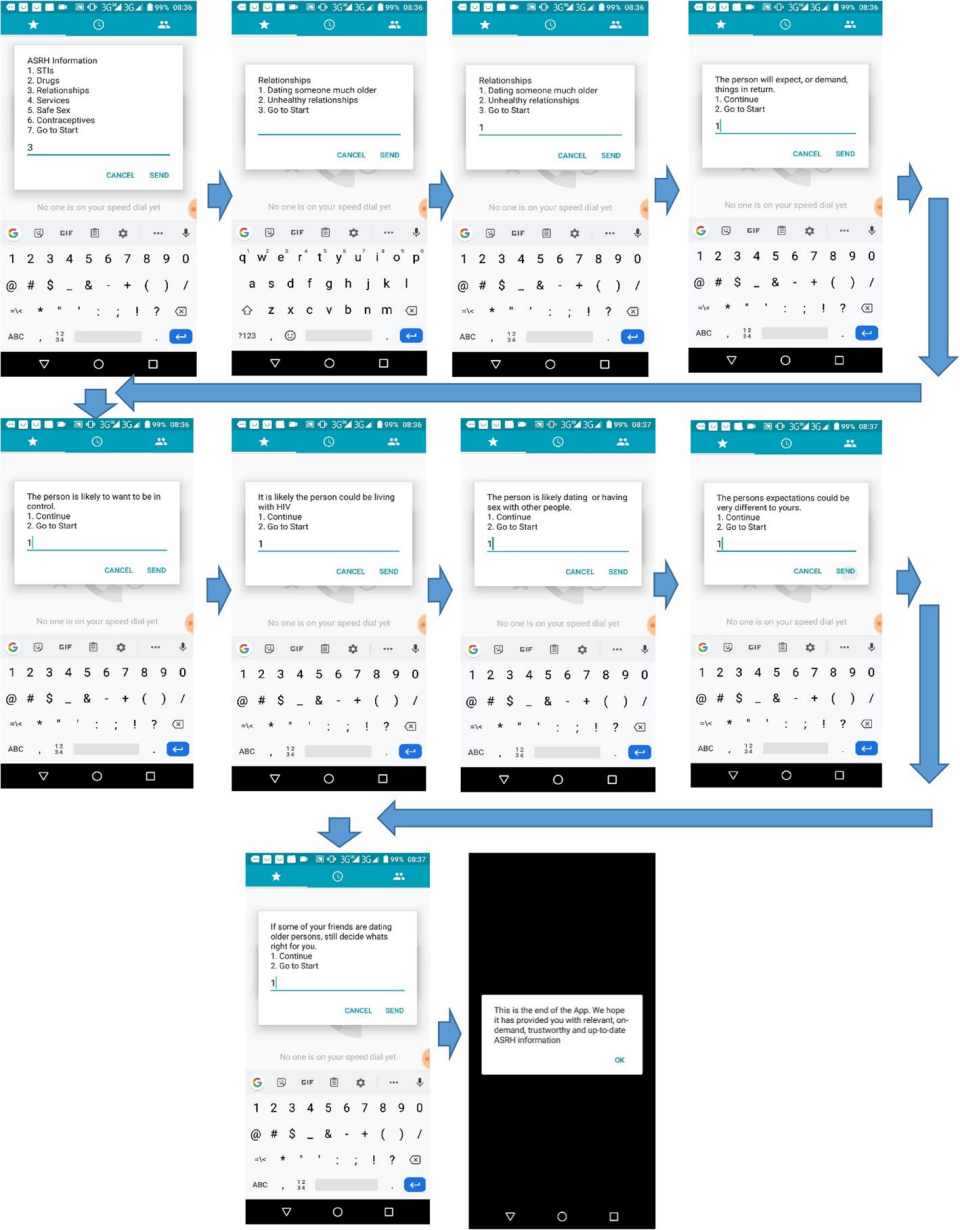
Accessing specific sexual reproductive health information in the USSD app

Adolescent users could select sexual reproductive health topics of interest and access information. Figure 3 shows how a user accessed content on sexual relationships.

### Alpha Testing

For the alpha testing, participants were required to dial a 7-digit USSD code on either a feature phone or smartphone., The users were then taken through authentication and selection of gender and age. Table 1 shows the demographic characteristics of the adolescents who were successfully followed up and had used the app at least once during the 1-month period of alpha usability testing. The median age of participants was 15 years for the under 18 and 18 years for the 18 and above group. All attended secondary school. Of the 38 adolescents enrolled, 12 were successfully followed up, only 9 had used the app at least once during the 1-month period. Only survey questions applicable to the USSD app were analyzed. Questions on app customization, sharing, app description on Playstore, buttons and icons were excluded.

**Table 1:** Demographic characteristics of alpha testing participants who used the app

	**Under 18 *n* (%)**	**18 and above *n* (%)**	***p*-value**
Participants	7 (77.8)	2 (22.2)	
Age median, (SD) (Range)	15.0, (0.53) (15 – 16)	18.0, (0.00) (18 – 18)	0.029
Gender			
Male	3 (100)	0 (0)	0.777
Female	4 (66.7)	2 (33.3)	
Education			
Primary	0 (0)	0 (0)	0.096
Secondary	7 (77.8)	2 (22.2)	
College	0 (0)	0 (0)	
University	0 (0)	0 (0)	
Occupation			
Student	7 (77.8)	2 (22.2)	0.096
None	0 (0)	0 (0)	

Participants feedback on engagement, functionality, aesthetics, and quality of information provided by the mobile app is shown in Table 2. The MARS was scored 1 to 5 with 1 being the lowest score and 5 the highest. The highest scores were attained on engagement of the app with a mean score of 4.4, and functionality with a mean score of 4.3. The MARS consisted of 15 items and the value for Cronbach’s Alpha for the survey was α = 0.83.

**Table 2:** Mobile application rating scale scores for the alpha testing

**Mobile Application Rating Scale Score**	**Mean (SD)** (Range)
Engagement	
Entertainment	4.1 (0.93) (2)
Interest	4.6 (1.00) (3)
Interactivity	4.2 (1.30) (4)
Target group	4.6 (0.73) (2)
** Mean score**	**4.4 (0.82)** **(2.7)**
Functionality score	
Performance	4.1 (1.27) (3)
Ease of use	3.8 (1.09) (4)
Navigation	4.8 (0.44) (1)
Gestural design	4.4 (0.73) (2)
** Mean score**	**4.3 (0.73)** **(2.3)**
Aesthetics score	
Visual appeal	3.3 (1.50) (4)
** Mean score**	**3.3 (1.50)** **(4)**
Information score	
Goals	4.0 (1.00) (2)
Quality of information	4.7 (0.70) (2)
Quantity of information	3.8 (1.30) (4)
** Mean score**	**4.1 (0.73)** **(2.4)**
** App quality mean Score**	**4.0 (0.74)** **(2.3)**

Under the aesthetics, information on layout, the “arrangement and size of buttons/icons” and graphics “the quality/resolution of graphics used for buttons/icons/menus/content” were excluded from the final analysis. The research team noted that these features were not applicable to the USSD app, the app contains no buttons, icons or graphics. Since no issues were identified during the alpha testing of the app, nothing was changed before the field testing.

### Field Usability Testing

Once the alpha usability test had been completed and performance of the app validated, field usability testing was carried out. Participants had to dial a 7-digit USSD code on either a feature phone or smartphone, before being taken through authentication and selection of gender and age. Once in the app, the users had a list of options to choose from based on their information needs. For the field testing, 146 adolescents were enrolled, 113 were followed-up, and 109 provided complete study data. Among these, 62 had used the app at least once in the 3-month period. The demographic characteristics of participants who used the app during the field usability testing are shown in Table 3. This p-values checked potential statistically significant difference in demographic characteristics between participants under 18 years and above 18 years. The mobile application rating scale scores for the field usability testing are shown in Table 4. Only survey questions applicable to a USSD app were analyzed. Questions on app customization, sharing, app description on Playstore, buttons and icons were excluded.

**Table 3:** Demographic characteristics of field testing participants who used the app

	**Below 18 *n* (%)**	**18 and above *n* (%)**	***p*-value**
Participants	33 (53.2)	29 (46.8)	
Age (median, SD) (Range)	17.0, (0.79) (15 – 17)	18.0, (0.44) (18 – 19)	1.102
Gender			
Male	17 (56.7)	13 (43.3)	0.786
Female	16 (50.0)	16 (50.0)	
Education			
Primary	6 (85.7)	1 (14.3)	0.111
Secondary	26 (51.9)	28 (48.1)	
None	1 (100)	0 (0)	
University	0 (0)	0 (0)	
Occupation			
Student	32 (52.5)	29 (47.5)	1.000
None	1 (100)	0 (0)

**Table 4:** Mobile application rating scale scores for the field testing

Mobile Application Rating Scale Score	Mean (SD) (Range)
Engagement	
Entertainment	3.8 (0.93) (4)
Interest	4.7 (0.46) (1)
Interactivity	4.0 (1.17) (4)
Target group	4.5 (0.56) (2)
** Mean score**	**4.3 (0.44)** **(1.7)**
Functionality score	
Performance	4.5 (0.97) (3)
Ease of use	4.2 (0.44) (1)
Navigation	4.8 (0.43) (2)
Gestural design	4.8 (0.35) (1)
** Mean score**	**4.6 (0.38)** **(1.7)**
Aesthetics score	
Visual appeal	4.3 (0.57) (2)
** Mean score**	**4.3 (0.57)** **(2)**
Information score	
Goals	4.1 (0.84) (2)
Quality of information	4.5 (0.71) (3)
Quantity of information	4.6 (1.11) (4)
** Mean score**	**4.4 (0.60)** **(2.3)**
** App quality mean Score**	**4.4 (0.31)** **(1.5)**

During the field usability testing, engagement of the app attained a mean score of 4.3 (0.44). The functionality mean score was 4.6 (0.38), with navigation and gestural design ratings within the functionality score attaining a mean score of 4.8 (0.43) and 4.8 (0.35) respectively. The overall mean score for information was 4.4 (0.31), with quantity of information attaining 4.6 (1.11) and quality of information 4.5 (0.71). The value for Cronbach’s Alpha for the field testing was α = 0.54. It is important to note that, 109 adolescents were successfully followed up after the 3-month period. However, only 62 had used the USSD app at least once. The usability testing interview was only administered to adolescent participants who had used the app at least once during the 3-month period. Table 5 shows participants feedback on the app’s characteristics. Only options selected by users are included in this table.

**Table 5:** User experience evaluation of the mobile application

App characteristics	All *n* (%)	Male *n* (%)	Female *n* (%)	*p*-value
**Times used app—median (SD)** **(Range)**	4 (9.9) (1 – 51)	3 (8.1) (1 – 37)	6 (10.9) (1 – 51)	0.534
**Is the app fun/entertaining to use?**				
Dull	1 (1.6)	1 (3.3)	0 (0)	0.708
Fun enough to entertain user	27 (43.6)	12 (40.0)	15 (46.9)	
Moderately fun and entertaining	15 (24.2)	8 (26.7)	7 (21.8)	
Highly entertaining and fun	19 (30.6)	9 (30.0)	10 (31.3)	
**Is the app interesting to use?**				
Mostly uninteresting	1 (1.6)	0 (0)	1 (3.1)	0.400
Moderately interesting	17 (27.4)	10 (33.3)	7 (21.9)	
Very interesting	44 (71.0)	20 (66.7)	24 (75.0)	
**Is the app content appropriate for you as an adolescent?**				
Acceptable but not targeted. May be inappropriate/unclear/confusing	2 (3.2)	1 (3.4)	1 (3.1)	0.974
Well targeted, with negligible issues	26 (41.9)	13 (43.3)	13 (40.6)	
Perfectly targeted, no issues found	34 (54.9)	16 (53.3)	18 (56.3)	
**How accurately do the app features and menus work?**				
Some functions work, but lagging or contains major technical problems	5 (8.1)	3 (10.0)	2 (6.2)	0.953
App works overall. Some technical problems need fixing	6 (9.7)	3 (10.0)	3 (9.4)	
Mostly functional with minor/negligible problems	6 (9.7)	3 (10.0)	3 (9.4)	
Perfect/timely response; no technical bugs found	45 (72.5)	21 (70.0)	24 (75.0)	
**How easy is it to learn how to use the app?**				
Easy to learn how to use the app	45 (72.6)	21 (70.0)	24 (75.0)	0.876
Able to use app immediately; intuitive; simple	17 (27.4)	9 (30.0)	8 (25.0)	
**Are interactions consistent and intuitive across all screens?**				
Mostly consistent/intuitive with negligible problems	9 (14.5)	3 (10.0)	6 (18.7)	0.537
Perfectly consistent and intuitive	53 (85.5)	27 (90.0)	26 (81.3)	
**Is app content correct, well written, and relevant to Adolescent Sexual Reproductive Health?**				
Barely relevant	2 (3.2)	2 (6.7)	0 (0)	0.237
Moderately relevant	2 (3.2)	0 (0)	2 (6.2)	
Relevant	23 (37.1)	12 (40.0)	11 (34.4)	
Highly relevant, appropriate, coherent, and correct	35 (56.5)	16 (53.3)	19 (59.4)	
**Is the content comprehensive and concise?**				
Minimal Information	4 (6.5)	2 (6.7)	2 (6.3)	0.237
Insufficient	1 (1.6)	0 (0)	1 (3.1)	
OK but not comprehensive or concise	3 (4.8)	3 (10.0)	0 (0)	
Comprehensive and concise; contains links to more information and resources	54 (87.1)	25 (83.3)	29 (90.6)	
**Would you recommend this app to people who might benefit from it?**				
There are very few people I would recommend this app to	3 (4.8)	0 (0)	3 (9.4)	0.274
There are several people whom I would recommend it to	6 (9.7)	3 (10.0)	3 (9.4)	
There are many people I would recommend this app to	19 (30.7)	8 (26.7)	11 (34.4)	
I would recommend this app to everyone	34 (54.8)	19 (63.3)	15 (46.8)	
**What is your overall rating of the app?**				
Average	6 (9.7)	6 (20.0)	0 (0)	**0.011**
Above average	11 (17.7)	6 (20.0)	5 (15.6)	
Best app	45 (72.6)	18 (60.0)	27 (84.4)	
**This app is likely to increase awareness of the importance of addressing Adolescent Sexual Reproductive Health?**				
Agree	34 (54.8)	20 (66.7)	14 (43.7)	0.119
Strongly agree	28 (45.2)	10 (33.3)	18 (56.3)	
**This app is likely to increase knowledge of Adolescent Sexual Reproductive Health?**				
Neutral	1 (1.6)	1 (3.3)	0 (0)	0.262
Agree	37 (59.7)	20 (66.7)	17 (53.1)	
Strongly agree	24 (38.7)	9 (30.0)	15 (46.9)	
**This app is likely to change attitudes toward improving Adolescent Sexual Reproductive Health?**				
Agree	43(69.4)	20 (66.7)	23 (71.9)	0.866
Strongly agree	19 (30.6)	10 (33.3)	9 (28.1)	
**This app is likely to increase intentions to address Adolescent Sexual Reproductive Health?**				
Agree	44 (81.0)	21 (70.0)	23 (71.9)	1.000
Strongly agree	18 (29.0)	9 (30.0)	9 (28.1)	
**Use of this app is likely to encourage further help seeking on Adolescent Sexual Reproductive Health?**				
Disagree	1 (1.6)	1 (3.3)	0 (0)	0.418
Agree	41 (66.1)	18 (60.0)	23 (71.9)	
Strongly agree	20 (32.3)	11 (36.7)	9 (28.1)	
**Use of this app is likely to reduce problems in Adolescent Sexual Reproductive Health?**				
Agree	41 (66.1)	20 (66.7)	21 (65.6)	1.000
Strongly agree	21 (33.9)	10 (33.3)	11 (34.4)	

The adolescents found the app entertaining, with 43.6% (27) of the users indicating the app was fun to use. Most of the users, 70.9% (44), found the app very interesting. The adolescents deemed the app content to be appropriately directed, with 54.8% (34) users indicating it was perfectly targeted. Only 3.2% (2) of the users felt the content was not well targeted or inappropriate.

On the accuracy of app features, 72.5% (45) of the adolescents felt the features were perfect and did not experience any bugs. The adolescents were able to learn how to use the app swiftly, with 72.6% (45) finding this easy. Regarding the app’s interaction, 85.5% (53) of the adolescents found the content consistent and intuitive across all screens.

Over 90% of the adolescents found the content provided in the app relevant to their sexual reproductive health needs. Gender differences were not significant. The content was comprehensive according to 87.1% (54) of the adolescents. Notably, at least one in every two participants, 54.8% (34), indicated they would recommend the app to other adolescents. The app was rated highly, with 72.6% (45) of users describing it as the ‘best app’ for providing adolescent sexual reproductive health information.

Although 45.2% (28) of users ‘strongly agreed’ the app could increase awareness of sexual reproductive health information, there were important gender differences within this category. While 66.7% (20) of male participants ‘agreed’ the app could increase awareness, less than half the female users, 43.7% (14), ‘agreed’. The majority of female participants, 56.3% (18), ‘strongly agreed’ the app could increase awareness. On increasing knowledge, 59.7% (37) of users ‘agreed’ the app was likely to increase knowledge on sexual reproductive health.

Users also felt that the app could change attitudes toward adolescent sexual reproductive health, with 69.4% (43) agreeing that a change in attitude would improve service provision and uptake. The app was envisioned to potentially increasing the uptake of interventions providing adolescent sexual reproductive health by 81.0% (44) of participants. On whether the app would encourage help-seeking behavior by adolescents on reproductive health issues, 66.1% (41) of participants agreed. A reduction in adolescent sexual reproductive health problems through use of the app was predicted by 66.1% (41) of participants.

A number of user experience evaluation components attained substantially different scores from male and female users. On the overall rating of the app, 72.6% (45) rated the app as the ‘best app’: of these, 84.4% (27) were female users compared to 60.0% (18) male, with a p-value = 0.011. On the USSD app increasing awareness of addressing adolescent sexual reproductive health, 56.3% (18) of the female users ‘strongly agreed’ compared to 33.3% (10) male users. On the likelihood of the app increasing knowledge on sexual reproductive health, 46.9% (15) of female users ‘strongly agreed’, but only 30.0% (9) of male users.

## Discussion

From a human-centered approach, we designed, developed and prototyped a USSD-based mobile app. Research has shown that adolescents want to be engaged in the design, development and prototyping of mobile apps that are used to monitor and manage their healthcare needs [31]. In this study, feedback from the adolescent participants was applied at every stage of the app development. The participants identified their sexual reproductive health information needs, alpha tested the initial app prototype and participated in field usability testing. Usability testing is a critical step in the development of an effective and engaging mobile app capable of impacting users’ health outcomes [32]. As their information and design requests were considered, the participants found that the USSD app effectively provided sexual reproductive health information.

In our study, the users found the mobile app engaging and easy to navigate. The information provided was of high quality, age-group specific and in the right quantity. Feroz et al. [33] found that mobile apps could be highly effective for providing adolescents with reproductive health information in resource-limited settings. This is due to the barriers adolescents have to contend with accessing SRH services at the health facilities. Mobile app users in Feroz’s study felt the intervention provided support and connectedness, especially if the app provided targeted communication containing new knowledge, reminders and/or suggestions about health issues [34]. Information provided by the USSD app in our study was found to be individualized, appropriate and relevant to the participants’ sexual reproductive health information needs.

The participants may have found that the USSD app enhanced confidentiality, a feature that influences how an adolescent will access sexual reproductive health information. Healthcare workers may take on a policing role guided by their own or socially-sanctioned standards. Technology-based reproductive health information sources, including the internet, are adolescents preferred options [35, 36]. The USSD app enabled the adolescents to access sexual reproductive health information in a confidential way. No audit trail was left on the mobile phone when the USSD sessions ended. Using the study mobile app, the adolescents could access information when needed/required without fear of being judged. It is important to note that there is no local installation, and no trace is kept in the phone after using the USSD app, any SRH information the adolescent accessed remains confidential improving the user’s confidence in the app.

The USSD mobile phone app had content that was accurate and its functionality was free of technical problems. The app was easy to use and the content consistent and intuitive across all interaction screens. As Steinberg et al. [37] demonstrated, mobile phone apps designed to provide adolescents with information should have functionality features that improve user experience and enable users to search content by topic, making it easier to access the required information. The adolescents in our study found the USSD app easy to use and quickly learnt how the app worked, navigating through the content with ease.

Adolescent participants in the study found the USSD app effective for providing sexual reproductive health information. Guilamo-Ramos et al. [38] showed that adolescent users are motivated to use technology-enabled access to reproductive health information due to its accessibility and wide coverage of topics that can be personalized to each user. The USSD app was deemed very appropriate to all the adolescents and could work toll-free on all types of phones. As the app met their sexual reproductive health information needs, the participants were prepared to recommend it to their peers.

The adolescents found the USSD app user-friendly. The USSD technology provides an interactive, user-friendly and simple tool for delivering health information using mobile phones. This technology has been used to build highly promising mobile phone-based clinical decision support systems for healthcare providers [39], USSD was also used to pilot a well-received mobile app enabling users to locate healthcare facilities in their vicinity [40].

As Canavarro et al. [3] determined, girls bear the greatest burden when unable to access accurate and up-to-date reproductive health information. Adolescent pregnancy can lead to dropping out of school, early marriage and/or rejection by family members. This may explain why more female participants in this study deemed the USSD app the ‘best app’ (p-value = 0.011) than male participants. Female participants also ‘strongly agreed’ the USSD app could increase awareness and knowledge of adolescent sexual reproductive health issues.

Although there are few apps in Playstore and Appstore on adolescent reproductive health, they only work on Android or iOS smartphones. Many adolescents in resource-limited settings including Kenya and specifically Kibra, our study site location may not easily access smartphones. This was confirmed by our qualitative work [17] during the exploratory stages of gathering the adolescents’ SRH information needs. USSD a low cost application that works on both smartphones and feature phones was the option of choice by the adolescent participants.

In alpha testing, research has found that a sample of 10 participants is able to identify over 80% of issues with an application at alpha testing stage [41]. In our study, 9 of the 38 participants were successfully followed-up attaining 90% of the minimum requirements. Adolescent research studies face unique challenges on recruitment, retention and follow-up [42]. In our study, after the alpha testing, we requested the ethics review board to offer the adolescents a gift voucher on successful follow-up. During the field testing, 113 (75%) adolescents were successfully followed-up. Innovate approaches need to be continuously undertaken to improve retention in adolescent studies.

In our study we used the MARS for the usability testing, other studies have used the System Usability Scale (SUS) to measure the usability of mobile apps. The SUS is a 10-question tool used to measure subjective usability [43]. In one setting that evaluated the reliability of a translated SUS assessing mobile apps, the reliability test showed a Cronbach alpha value of 0.85 indicating the SUS is a reliable tool for usability assessment of mobile apps [44]. The MARS measures a significant number of mobile apps quality dimensions and has also been validated in more mhealth apps [45].

Usability testing has also been used to test mhealth apps for other health needs and conditions. In one study, usability testing was used to evaluate an mhealth app that provided information on how to manage chronic conditions to persons living with HIV. From the usability testing, the researchers were able to get useful feedback that made the mhealth app usable and ready for future efficacy testing. [46]. In another study, usability testing evaluated mhealth apps in paediatric obesity. The findings of the study identified the importance of thorough evaluation and collection of evidence in mhealth apps for best practice [47].

### Limitations

Although 62 adolescent participants used the app at least one during the 3-month period meeting the minimum sample required, the study findings could have had more confidence if all participants in the intervention group used the app. This study targeted adolescents that could access a mobile phone, adolescents not able to access a mobile phone were screened out. Further research could be carried out to find out how adolescents not able to access a mobile phone could access SRH information.

On participant selection, adolescents were mobilized from youth program venues where 15 to 19 year olds visited. Adolescents who did not visit these places were not enrolled creating a potential selection bias. Relating to the selection bias, what was identified as SRH information needed by the adolescents was based on the sample of adolescents enrolled. A subset of adolescents in Kibra that was not enrolled could have had different SRH information needs. Content provided in the USSD app may have been biased to the needs of enrolled participants. Studies exploring multiple participant mobilization strategies need to be explored to ensure all adolescent get a chance to participate in research studies thus informing interventions.

Although the USSD technology works on feature phones, most feature phone devices have smaller display screens. Adolescents using feature phones may have faced challenges of being able to view the information displayed on their screens. One way we attempted to address this was to keep the statements short and precise. The most effective solution to this limitation is for the adolescents to use a device with a larger display screen.

## Conclusions

Adolescents need information on sexual reproductive health, they also want to be meaningfully involved in the design and development process of interventions intended to meet their SRH information needs. Being involved at every stage of the app design and development in this study, participants found both the content and USSD app very appropriate. The USSD app worked on their feature phones and they could access the services toll-free. Their privacy was protected by the absence of an audit trail.

The USSD mobile phone technology is ideal for resource-limited settings, as users in these areas may only be able to access feature phones, have to share a phone and/or be unable to pay for services. The USSD technology works seamlessly on both feature phones and smartphones. No information is saved on the phone when using USSD, thus maintaining confidentiality even on shared phones. The service can be pre-paid by the provider. For future work, with availability of resources, we aim to scale-up prototyping and testing the mobile app with adolescents in different parts of Kenya and the Africa region. Expanding the USSD-based technology providing SRH information to adolescents living in underprivileged settings could have positive life-long impact on the adolescents’ reproductive health.

## Supplementary Information

**Additional file 1: Appendix A**. Paper-based version of the USSD app content. **Appendix B**. A customized mobile application rating scale.

## Data Availability

The datasets used and/or analyzed during the current study are available from the corresponding author (pmacharia@uoc.edu) on reasonable request.

## Abbreviations

HCD: Human-centered design
MARS: Mobile application rating scale
PIN: Personal identification number
SD: Standard deviation
SRH: Sexual reproductive health
STIs: Sexually-transmitted infections
WHO: World Health Organization
USSD: Unstructured Supplementary Service Data
