# Supplementary material for: Developing an Unstructured Supplementary Service Data-based mobile phone app to provide adolescents with sexual reproductive health information: a human-centered design approach
Source: BMC Med Res Methodol. 2022 Aug 4;22:213. doi: 10.1186/s12874-022-01689-4 (PMC9351084; doi:10.1186/s12874-022-01689-4)
Supplement: Supplementary file 1 — Additional file 1: Appendix A. Paper-based version of the USSD app content. Appendix B. A customized mobile application rating scale. [file 12874_2022_1689_MOESM1_ESM.docx]

**Appendix A: Paper-based version of the USSD app content**

| **No.** | **Question** | **Response** |
| --- | --- | --- |
| 1 | Age | 1. 15 (go to 3a) 2. 16 (go to 3a) 3. 17 (go to 3a) 4. 18 (go to 3b) 5. 19 (go to 3b) |
| 2 | Gender | 1. Male 2. Female |
| 3 | **ASRH Information** |  |
| 3a | List of options (Age 15 - 17) | 1. STIs Information (go to 4) 2. Abstain from Sex (go to 13) 3. Abstain from Drugs (go to 3c) 4. Sexual relationship (go to 6) 5. Adolescent friendly services (go to 18) |
| 3b | List of options (Age 18 and above) | 1. STIs Information (go to 4) 2. Contraceptives (go to 5) 3. Safe Sex (go to 13) 4. Abstain from Drugs (go to 3c) 5. Sexual Relationships (go to 6) 6. Adolescent friendly services (go to 18) |
| 3c | Abstain from drugs | 1. Drug use screening 2. Drugs and sex |
| 4 | STI Information | 1. STI Symptoms (go to 7) 2. Talking about HIV (go to 10) |
| 5 | Contraceptives | 1. Options (go to 11) 2. Sex with no condom (go to 12) |
| 6 | Sexual relationships | 1. Dating someone much older (go to 14) 2. Signs of unhealthy relationships (go to 15) |
| **STIs Information** | | |
| 7 | Symptomatic STI Screening | 1. Female (go to 8) 2. Male (go to 9) |
| 8 | Symptomatic STI Screening  Females |  |
| 8a | Vaginal itching | 1. Yes 2. No |
| 8b | Vaginal discharge | 1. Yes 2. No |
| 8c | Painful urination | 1. Yes 2. No |
| 8d | Increased urinary urgency | 1. Yes 2. No |
| 8e | Pain in the lower abdomen | 1. Yes 2. No |
| 8f | Pain during sexual intercourse | 1. Yes 2. No |
| 8g | Vaginal bleeding (not periods) | 1. Yes 2. No |
| 8h | Genital blisters (swellings filled with fluid) | 1. Yes 2. No |
| 8i | Genital ulcer or wound | 1. Yes 2. No |
| 8j | You need further assessment (If “Yes” in any of 8a – 8i go to 18) |  |
| 9 | Symptomatic STI Screening Males |  |
| 9a | Penile/urethral discharge | 1. Yes 2. No |
| 9b | Painful urination | 1. Yes 2. No |
| 9c | Increased urgency | 1. Yes 2. No |
| 9d | Pain in the lower abdomen | 1. Yes 2. No |
| 9e | Swollen/painful testicles | 1. Yes 2. No |
| 9f | Pain during sexual intercourse | 1. Yes 2. No |
| 9g | Genital blisters (swellings filled with fluid) | 1. Yes 2. No |
| 9h | Genital ulcer or wound | 1. Yes 2. No |
| 9i | You need further assessment (If “Yes” in any of 9a – 9h go to 18) |  |
| **10. Talking about HIV with a partner** | | |
| 10a | You don’t have to apologize because you are living with HIV. | 1. Continue |
| 10b | Have some information on hand to share with them. | 1. Continue |
| 10c | Think of the best time to tell them, when you are relaxed and have enough time. | 1. Continue |
| 10d | Find a private place you are less likely to be interrupted. | 1. Continue |
| 10e | Whatever your partner’s first reaction, it could change over time. | 1. Continue |
| 10f | It is important your partner is tested. Meanwhile, if in a sexual relationship practice safer sex/use condoms. | 1. Continue |
| 10g | Fear and stigma could stir up very strong emotions, if needs be, see a counselor. | 1. Continue |
| 10h | Your status may make some people afraid or judgmental. | 1. Continue 2. Main |
| **Contraceptives** | | |
| 11 | **Options for contraception** |  |
| 11a | Many contraceptives only prevent pregnancy. | 1. Continue |
| 11b | Consider how long lasting you want it to be; if pills or injection, are you able to remember to take it or get injection on time. | 1. Continue |
| 11c | It is a good idea to talk about options with your partner. | 1. Continue |
| 11d | **Condoms** prevents both pregnancy and STIs. | 1. Continue |
| 11e | The **contraceptive pill** is taken by women to prevent pregnancy but has no protection from STIs. | 1. Continue |
| 11f | An **implant** for women prevents pregnancy and lasts up to four years but has no protection from STIs. | 1. Continue |
| 11g | **Injections** for women prevent pregnancy but have no protection from STIs. | 1. Continue 2. Main |
| 12 | **Sex with no condom** | 1. Continue |
| 12a | One of the only protections from STIs and pregnancy. | 1. Continue |
| 12b | Talk about it beforehand, be clear on what you both want. | 1. Continue |
| 12c | A partner asking for sex without a condom is a sign they are not taking care of themselves or you. | 1. Continue |
| 12d | Remember that you should never use a condom more than once. | 1. Continue |
| 12e | Neither should you double up, this creates friction and condoms could break. | 1. Continue |
| 12f | Condoms usually come pre-lubricated, but extra lubrication reduces STI risk. | 1. Continue |
| 12g | If using latex condoms, make sure you use a water-based lube. | 1. Continue 2. Main |
| **13. Abstain from sex** | | |
| 13a | You can say no at any time. | 1. Continue |
| 13b | Being in a relationship does not you’re your partner the right to do anything they want to you. | 1. Continue |
| 13c | You can change your mind about sex at any time. | 1. Continue |
| 13d | The legal age to have sex in Kenya is 18. | 1. Continue |
| 13e | Sexual contact without consent is wrong whatever the age of people involved. | 1. Continue |
| 13f | It can be difficult to say no in the heat of the moment – let your partner know your wishes beforehand. | 1. Continue 2. Main |
| **Sexual Relationships** | | |
| 14 | **Dating someone much older than you** | 1. Continue |
| 14a | The person will expect, or demand, things in return. | 1. Continue |
| 14b | The person is likely to want to be in control. | 1. Continue |
| 14c | It is likely the person could be living with HIV. | 1. Continue |
| 14d | The person is likely dating/having sex with other people. | 1. Continue |
| 14e | The person’s expectations could be very different to yours. | 1. Continue |
| 14f | If some of your friends are dating older people, you must still decide what’s right for you. | 1. Continue 2. Main |
| 15 | **Signs of unhealthy relationships** |  |
| 15a | Partner attempts to exercise control and power over you. | 1. Continue |
| 15b | Partner pressures you to send intimate photo messages that you don’t want to. | 1. Continue |
| 15c | You don’t feel able or willing to communicate with your partner. | 1. Continue |
| 15d | Your partner is being repeatedly dishonest. | 1. Continue |
| 15e | They call you names, constantly question and criticize your choices and decisions. | 1. Continue |
| 15f | You don’t feel you have any control over whether to use contraceptives. | 1. Continue |
| 15g | You don’t feel able to go out and see your friends without your partner being angry or sad or jealous. | 1. Continue |
| 15h | You are made to feel guilty for the choices that you make. | 1. Continue 2. Main |
| **Abstain from Drugs** | | |
| 16 | Adolescent Screening tool for drug use disorders. |  |
| Part A | |  |
| 16a | During the PAST 12 MONTHS, did you: Drink any alcohol? | 1. Yes 2. No |
| 16b | During the PAST 12 MONTHS, did you: Smoke any marijuana? | 1. Yes 2. No |
| 16c | During the PAST 12 MONTHS, did you: Use anything else to get high? | 1. Yes 2. No |
| If “Yes” in any of 16a – 16c go to Part B | |  |
| Part B | |  |
| 16d | Have you ever ridden in a CAR driven by someone who had been using alcohol or drugs? | 1. Yes 2. No |
| 16e | Do you ever use alcohol or drugs to RELAX, feel better about yourself, or fit in? | 1. Yes 2. No |
| 16f | Do you ever use alcohol or drugs while you are by yourself, or ALONE? | 1. Yes 2. No |
| 16g | Do you ever FORGET things you did while using alcohol or drugs? | 1. Yes 2. No |
| 16h | Do your FAMILY or FRIENDS ever tell you that you should cut down on your drinking or drug use? | 1. Yes 2. No |
| 16i | Have you ever got into TROUBLE while you were using alcohol or drugs? | 1. Yes 2. No |
| 16j | You need additional assessment.(If “Yes” for two or more question 16a -16i)(Go to 18e) |  |
| 17 | Risks of mixing drugs and sex? | 1. Continue |
| 17a | Forget to use a condom. | 1. Continue |
| 17b | Not able to consent for sex. | 1. Continue |
| 17c | Unaware of someone spiking your drink. | 1. Continue |
| 17d | Engaging in more risky sexual activities. | 1. Continue 2. Main |
| **Information on youth friendly services** | | |
| 18 | The following are youth friendly services in your neighborhood | 1. Facility A (go to 18a) 2. Facility B (go to 18b) 3. Facility C (go to 18c) 4. Facility D (go to 18d) |
| 18a | Contact details of facility A |  |
| 18b | Contact details of facility B |  |
| 18c | Contact details of facility C |  |
| 18d | Contact details of facility D |  |
| 18e | Contact details of facility E  (Drug use screening) |  |

**Appendix B: A customized mobile application rating scale**

**App Classification**

**App Quality Ratings**

The Rating scale assesses app quality on four dimensions. All items are rated on a 5-point scale from “1. Inadequate” to “5. Excellent”. Circle the number that most accurately represents the quality of the app component you are rating. Please use the descriptors provided for each response category.

**SECTION A**

Engagement – *fun, interesting, customizable, interactive (e.g., sends alerts, messages, reminders, feedback, enables sharing), well-targeted to audience.*

1. Entertainment: *Is the app fun/entertaining to use? Does it use any strategies to increase engagement through entertainment (e.g., through gamification)?*

1 Dull, not fun or entertaining at all

2 Mostly boring

3 OK, fun enough to entertain user for a brief time (< 5 minutes)

4 Moderately fun and entertaining, would entertain user for some time (5-10 minutes total)

5 Highly entertaining and fun, would stimulate repeat use

2. Interest: *Is the app interesting to use? Does it use any strategies to increase engagement by presenting its content in an interesting way?*

1 Not interesting at all

2 Mostly uninteresting

3 OK, neither interesting nor uninteresting; would engage user for a brief time (< 5 minutes)

4 Moderately interesting; would engage user for some time (5-10 minutes total)

5 Very interesting, would engage user in repeat use

3. Customization: *Does it provide/retain all necessary settings/preferences for apps features (e.g., sound, content, notifications, etc.)?*

1 Does not allow any customization or requires setting to be input every time

2 Allows insufficient customization limiting functions

3 Allows basic customization to function adequately

4 Allows numerous options for customization

5 Allows complete tailoring to the individual’s characteristics/preferences, retains all settings

4. Interactivity: *Does it allow user input, provide feedback, contain prompts (reminders, sharing options, notifications, etc.)? Note: these functions need to be customizable and not overwhelming in order to be perfect*.

1 No interactive features and/or no response to user interaction

2 Insufficient interactivity, or feedback, or user input options, limiting functions

3 Basic interactive features to function adequately

4 Offers a variety of interactive features/feedback/user input options

5 Very high level of responsiveness through interactive features/feedback/user input options

5. Target group: *Is the app content (visual information, language, design) appropriate for your target audience?*

1 Completely inappropriate/unclear/confusing

2 Mostly inappropriate/unclear/confusing

3 Acceptable but not targeted. May be inappropriate/unclear/confusing

4 Well targeted, with negligible issues

5 Perfectly targeted, no issues found

**A. Engagement mean score =**

**SECTION B**

Functionality *– app functioning, easy to learn, navigation, flow logic, and gestural design of app*

6. Performance: *How accurately/fast do the app features (functions) and components (buttons/menus) work?*

1 App is broken; no/insufficient/inaccurate response (e.g., crashes/bugs/broken features, etc.)

2 Some functions work, but lagging or contains major technical problems

3 App works overall. Some technical problems need fixing/slow at times

4 Mostly functional with minor/negligible problems

5 Perfect/timely response; no technical bugs found/contains a ‘loading time left’ indicator

7. Ease of use: *How easy is it to learn how to use the app; how clear are the menu labels/icons and instructions?*

1 No/limited instructions; menu labels/icons are confusing; complicated

2 Useable after a lot of time/effort

3 Useable after some time/effort

4 Easy to learn how to use the app (or has clear instructions)

5 Able to use app immediately; intuitive; simple

8. Navigation: *Is moving between screens logical/accurate/appropriate/ uninterrupted; are all necessary screen links present?*

1 Different sections within the app seem logically disconnected and random/confusing/navigation is difficult

2 Usable after a lot of time/effort

3 Usable after some time/effort

4 Easy to use or missing a negligible link

5 Perfectly logical, easy, clear and intuitive screen flow throughout, or offers shortcuts

9. Gestural design: *Are interactions (taps/swipes/pinches/scrolls) consistent and intuitive across all components/screens?*

1 Completely inconsistent/confusing

2 Often inconsistent/confusing

3 OK with some inconsistencies/confusing elements

4 Mostly consistent/intuitive with negligible problems

5 Perfectly consistent and intuitive

**B. Functionality mean score =**

**SECTION C**

Aesthetics *– graphic design, overall visual appeal, color scheme, and stylistic consistency*

10. Layout: *Is arrangement and size of buttons/icons/menus/content on the screen appropriate or zoomable if needed?*

1 Very bad design, cluttered, some options impossible to select/locate/see/read device display not optimized

2 Bad design, random, unclear, some options difficult to select/locate/see/read

3 Satisfactory, few problems with selecting/locating/seeing/reading items or with minor screen- size problems

4 Mostly clear, able to select/locate/see/read items

5 Professional, simple, clear, orderly, logically organized, device display optimized. Every design component has a purpose

11. Graphics: *How high is the quality/resolution of graphics used for buttons/icons/menus/content?*

1 Graphics appear amateur, very poor visual design – disproportionate, completely stylistically inconsistent

2 Low quality/low resolution graphics; low quality visual design – disproportionate, stylistically inconsistent

3 Moderate quality graphics and visual design (generally consistent in style)

4 High quality/resolution graphics and visual design – mostly proportionate, stylistically consistent

5 Very high quality/resolution graphics and visual design - proportionate, stylistically consistent throughout

12. Visual appeal: *How good does the app look?*

1 No visual appeal, unpleasant to look at, poorly designed, clashing/mismatched colors

2 Little visual appeal – poorly designed, bad use of color, visually boring

3 Some visual appeal – average, neither pleasant, nor unpleasant

4 High level of visual appeal – seamless graphics – consistent and professionally designed

5 As above + very attractive, memorable, stands out; use of color enhances app features/menus

**C. Aesthetics mean score =**

**SECTION D**

Information – *Contains high quality information (e.g., text, feedback, measures, references) from a credible source. Select N/A if the app component is irrelevant.*

13. Accuracy of app description (in app store): *Does app contain what is described?*

1 Misleading. App does not contain the described components/functions. Or has no description

2 Inaccurate. App contains very few of the described components/functions

3 OK. App contains some of the described components/functions

4 Accurate. App contains most of the described components/functions

5 Highly accurate description of the app components/functions

14. Goals: *Does app have specific, measurable, and achievable goals (specified in app store description or within the app itself)? N/A Description does not list goals, or app goals are irrelevant to research goal (e.g., using a game for educational purposes)*

1 App has no chance of achieving its stated goals

2 Description lists some goals, but app has very little chance of achieving them

3 OK. App has clear goals, which may be achievable.

4 App has clearly specified goals, which are measurable and achievable

5 App has specific and measurable goals, which are highly likely to be achieved

15. Quality of information: *Is app content correct, well written, and relevant to the goal/topic of the app? N/A There is no information within the app*

1 Irrelevant/inappropriate/incoherent/incorrect

2 Poor. Barely relevant/appropriate/coherent/may be incorrect

3 Moderately relevant/appropriate/coherent/and appears correct

4 Relevant/appropriate/coherent/correct

5 Highly relevant, appropriate, coherent, and correct

16. Quantity of information: *Is the extent coverage within the scope of the app; and comprehensive but concise? N/A There is no information within the app*

1 Minimal or overwhelming

2 Insufficient or possibly overwhelming

3 OK but not comprehensive or concise

4 Offers a broad range of information, has some gaps or unnecessary detail; or has no links to more information and resources

5 Comprehensive and concise; contains links to more information and resources

17. Visual information: *Is visual explanation of concepts – through charts/graphs/images/videos, etc. – clear, logical, correct? N/A There is no visual information within the app (e.g., it only contains audio, or text)*

1 Completely unclear/confusing/wrong or necessary but missing

2 Mostly unclear/confusing/wrong

3 OK but often unclear/confusing/wrong

4 Mostly clear/logical/correct with negligible issues

5 Perfectly clear/logical/correct

18. Credibility: *Does the app come from a legitimate source (specified in app store description or within the app itself)?*

1 Source identified but legitimacy/trustworthiness of source is questionable (e.g., commercial business with vested interest)

2 Appears to come from a legitimate source, but it cannot be verified (e.g., has no webpage)

3 Developed by small NGO/institution (hospital/center, etc.) /specialized commercial business, funding body

4 Developed by government, university or as above but larger in scale

5 Developed using nationally competitive government or research funding (e.g., Australian Research Council, NHMRC)

19. Evidence base: *Has the app been trialed/tested; must be verified by evidence (in published scientific literature)? N/A The app has not been trialed/tested*

1 The evidence suggests the app does not work

2 App has been trialed (e.g., acceptability, usability, satisfaction ratings) and has partially positive outcomes in studies that are not randomized controlled trials (RCTs), or there is little or no contradictory evidence.

3 App has been trialed (e.g., acceptability, usability, satisfaction ratings) and has positive outcomes in studies that are not RCTs, and there is no contradictory evidence.

4 App has been trialed and outcome tested in 1 – 2 RCTs indicating positive results

5 App has been trialed and outcome tested in > 3 high quality RCTs indicating positive results

**D. Information mean score = ***

** Exclude questions rated as “N/A” from the mean score calculation.*

SECTION E: App subjective quality

1. Would you recommend this app to people who might benefit from it?

1 Not at all I would not recommend this app to anyone

2 There are very few people I would recommend this app to

3 Maybe There are several people I would recommend this app to

4 There are many people I would recommend this app to

5 Definitely I would recommend this app to everyone

2. How many times do you think you would use this app in the next 12 months if it was relevant to you?

1 None

2 1 – 2

3 3 – 10

4 10 – 50

5 >50

3. Would you pay for this app?

1 No

3 Maybe

5 Yes

4. What is your overall rating of the app?

1 One of the worst apps I’ve used

2 Below Average App

3 Average App

4 Above Average App

5 One of the best apps I've used

**Scoring**

**App quality scores for**

**SECTION**

A: Engagement Mean Score =

B: Functionality Mean Score =

C: Aesthetics Mean Score =

D: Information Mean Score =

App quality mean Score =

App subjective quality Score =

**App-specific**

These added items can be adjusted and used to assess the perceived impact of the app on the user’s knowledge, attitudes, and intentions to change, as well as the likelihood of actual change in the target health behavior.

**SECTION F**

1. Awareness: *This app is likely to increase awareness of the importance of addressing Adolescent Sexual Reproductive Health*

Strongly disagree 1 2 3 4 5 Strongly Agree

2. Knowledge: *This app is likely to increase knowledge/understanding of Adolescent Sexual Reproductive Health*

Strongly disagree 1 2 3 4 5 Strongly Agree

3. Attitudes: *This app is likely to change attitudes toward improving Adolescent Sexual Reproductive Health*

Strongly disagree 1 2 3 4 5 Strongly Agree

4. Intention to change: *This app is likely to increase intentions/motivation to address Adolescent Sexual Reproductive Health*

Strongly disagree 1 2 3 4 5 Strongly Agree

5. Help seeking: *Use of this app is likely to encourage further help seeking for Adolescent Sexual Reproductive Health*

Strongly disagree 1 2 3 4 5 Strongly Agree

6. Behavior change: *Use of this app is likely decrease problems in Adolescent Sexual Reproductive Health*

Strongly disagree 1 2 3 4 5 Strongly Agree
